# Endogenous Taurine Downregulation Is Required for Renal Injury in Salt-Sensitive Hypertensive Rats via CBS/H_2_S Inhibition

**DOI:** 10.1155/2021/5530907

**Published:** 2021-08-25

**Authors:** Pan Huang, Yaqian Huang, Boyang Lv, Heng Zhang, Jia Liu, Guosheng Yang, Yinghong Tao, Dingfang Bu, Guang Wang, Junbao Du, Hongfang Jin

**Affiliations:** ^1^Department of Pediatrics, Peking University First Hospital, Beijing 100034, China; ^2^Department of Endocrinology, Beijing Chaoyang Hospital, Capital Medical University, Beijing 100020, China; ^3^Animal Center, Peking University First Hospital, Beijing 100034, China; ^4^Research Center, Peking University First Hospital, Beijing 100034, China; ^5^Key Laboratory of Molecular Cardiology, Ministry of Education, Beijing, China

## Abstract

Although taurine is known to exert an antihypertensive effect, it is unclear whether it is involved in the mechanism for hypertension-related target organ injury. To reveal the role of endogenous taurine in renal injury formation during salt-sensitive hypertension and clarify its mechanisms, both salt-sensitive Dahl rats and salt-resistant SS-13BN rats were fed a high-salt diet (8% NaCl) and given 2% taurine for 6 weeks. Rat systolic blood pressure (SBP) was measured by the tail-cuff method and artery catheterization. Kidney ultrastructure was observed under an electron microscope. Taurine content and mRNA and protein levels of taurine synthases, cysteine dioxygenase type 1 (CDO1) and cysteine sulfinic acid decarboxylase (CSAD), were decreased in Dahl rats fed a high-salt diet. However, taurine supplementation and the resulting increase in renal taurine content reduced the increased SBP and improved renal function and structural damage in high-salt diet-fed Dahl rats. In contrast, taurine did not affect SS-13BN SBP and renal function and structure. Taurine intervention increased the renal H_2_S content and enhanced cystathionine-*β*-synthase (CBS) expression and activity in Dahl rats fed a high-salt diet. Taurine reduced the renin, angiotensin II, and aldosterone contents and the levels of oxidative stress indices in Dahl rat renal tissues but increased antioxidant capacity, antioxidant enzyme activity, and protein expression. However, taurine failed to achieve this effect in the renal tissue of SS-13BN rats fed a high-salt diet. Pretreatment with the CBS inhibitor HA or renal CBS knockdown inhibited H_2_S generation and subsequently blocked the effect of taurine on renin, superoxide dismutase 1 (SOD1), and superoxide dismutase 2 (SOD2) levels in high-salt-stimulated Dahl renal slices. In conclusion, the downregulation of endogenous taurine production resulted in a decrease in the renal CBS/H_2_S pathway. This decrease subsequently promoted renin-angiotensin-aldosterone system (RAAS) activation and oxidative stress in the kidney, ultimately contributing to renal injury in salt-sensitive Dahl rats.

## 1. Introduction

The kidney is the main target of organ damage due to hypertension. A chronic increase in arterial pressure leads to a decrease in the glomerular filtration rate, proteinuria, and ultimately renal failure [1]. Chronic kidney disease, which is caused by hypertension, is a powerful independent risk factor for adverse cardiovascular outcomes [2]. However, the pathogenesis of hypertensive renal injury is still poorly understood. Dietary habits are closely related to the incidence of hypertension [3]. Up to 50% of patients with essential hypertension have high salt-induced hypertension, also known as salt-sensitive hypertension [4]. Notably, salt-sensitive hypertensive patients are more likely to develop end-stage renal disease compared with salt-resistant hypertensive patients [5]. High salt intake also directly causes renal damage [6]. The activation of the renin-angiotensin-aldosterone system (RAAS), oxidative stress, and inflammation are all related to high salt-induced renal damage [7]. However, the mechanism for endogenous regulation of salt-sensitive hypertensive renal injury has not been fully elucidated.

Taurine is a nonprotein free amino acid produced endogenously from cysteine by the oxidation of cysteine dioxygenase (CDO) and decarboxylation of cysteine sulfinic acid decarboxylase (CSAD) [8]. Taurine has a variety of biological effects, including the inhibition of RAAS activation, antioxidant stress, and vasodilation, promotion of water and sodium metabolism, and suppression of sympathetic nerve excitation [9, 10]. Taurine supplementation can decrease blood pressure in various hypertensive animal models, including L-NAME-induced hypertensive rats [11], stroke-prone spontaneously hypertensive rats [12], cyclosporine A-induced hypertensive rats [13], and DOCA-salt rats [14]. Oral taurine has been shown to significantly lower blood pressure and protect vascular function and responsiveness in clinical hypertensive patients [15]. These studies suggest that taurine is of great potential value in the mechanism and clinical treatment of hypertension. However, the changes in endogenous taurine during high salt-induced hypertensive renal injury and the possible mechanisms by which it impacts renal injury are unclear and warrant further exploration.

In the past, hydrogen sulfide (H_2_S) was considered a waste gas. However, over the past two decades, it has been found that there is a complete H_2_S-generating pathway in mammals [16]. L-cysteine produces H_2_S under the catalysis of cystathionine-*β*-synthase (CBS) and cystathionine-*γ*-lyase (CSE) [17]. Moreover, 3-mercaptopyruvate, which can be produced either from L-cysteine catalyzed by cysteine aminotransferase or from D-cysteine catalyzed by D-amino-acid oxidase (DAO), also generates H_2_S under the catalysis of mercaptopyruvate sulfurtransferase (MPST) [17–19]. CBS, CSE, and MPST are all expressed in the brain, heart, lung, artery, and kidney [17, 18, 20]. An increasing number of studies have revealed that endogenous H_2_S is an important gasotransmitter and exerts various physiological and pathophysiological effects [17–22]. Endogenous H_2_S generation was reportedly downregulated in several hypertensive animal models including spontaneously hypertensive rats (SHRs) [23] and models of salt-sensitive hypertension [24], L-NAME-induced hypertension [25], and two-kidney one-clip hypertension [26]. Furthermore, supplementation of the H_2_S donor sodium hydrosulfide (NaHS) significantly alleviated hypertension [27] and suppressed renal oxidative stress [28, 29], implying that the downregulated endogenous H_2_S pathway might be important in the mechanism underlying hypertensive renal damage. Sun et al. [15] found that taurine could reduce the blood pressure of patients with prehypertension, improve vasodilation, and impact the plasma H_2_S content. However, whether endogenous taurine participates in the development of salt-sensitive hypertensive renal injury through regulating the H_2_S-generating pathway remains to be clarified.

In this study, the change in and role of the endogenous taurine pathway during salt-sensitive hypertensive renal damage and the role of the renal H_2_S-generating pathway in controlling renal damage via taurine were examined.

## 2. Materials and Methods

### 2.1. Materials

High-salt (HS) feed containing 8% sodium chloride (NaCl) was purchased from Beijing Keao Xieli Feed Company (Beijing, China), taurine was purchased from Beijing Puboxin Biotechnology Company (Beijing, China), and horseradish peroxidase-labeled secondary antibodies, NaHS, and the CBS inhibitor hydroxylamine hydrochloride (HA) were purchased from Sigma-Aldrich (St. Louis, MO, USA) [30]. Antibodies against CBS , MPST, gp91phox, p47phox, p22phox and CD31 were purchased from Santa Cruz (Dallas, TX, USA); CSE antibody was purchased from Sigma-Aldrich; antibodies against CSAD, CDO1, CD31 and renin were purchased from Abcam (Waltham, MA, USA); *β*-actin antibody was purchased from Beijing Zhongshan Golden Bridge Biotechnology Co., Ltd. (Beijing, China); GAPDH antibody was from Shanghai Kangcheng Biological Engineering Company (Shanghai, China), and antibodies against SOD1 and SOD2 were purchased from Enzo Life (Farmingdale, NY, USA).

### 2.2. Animal Experiments

Animal experiments were approved by the Animal Research Ethics Committee of Peking University First Hospital (approval number: j201205) and strictly abided by the regulations of the Animal Experiment Center of Peking University First Hospital and the laboratory animal welfare rules. Male salt-sensitive (Dahl) and salt-resistant (SS-13BN) rats (*n* = 30 per group, 5 weeks old, and weighing 180–200 g) were obtained from Beijing Vital River Laboratory Animal Technology Company (Beijing, China). These two types of rats were further randomly divided into three groups (*n* = 10 per group) and given a normal diet containing 0.5% NaCl (Dahl+normal salt (NS), SS-13BN+NS), an HS diet containing 8% NaCl [31] (Dahl+HS, SS-13BN+HS), or an HS diet combined with 2% taurine [11] in drinking water (Dahl+HS+Tau, SS-13BN+HS+Tau) for 6 weeks.

### 2.3. Blood Pressure Measurement

The tail arterial pressure of awake rats was measured using an intelligent noninvasive blood pressure monitor [32]. After the experiment, rat systolic blood pressure (SBP) was also determined using the carotid artery catheter method [33]. Briefly, the rat was anesthetized with 25% urethane and then intubated through the right common carotid artery. Rat SBP was recorded using a PT-100 biological blood pressure sensor and a BL-420F multichannel physiological signal acquisition and recording system (Chengdu Techman Instrument Co., Ltd., Sichuan, China).

### 2.4. Determination of Biochemical Indicators

After the experiment, 24-h urine samples were collected using a metabolic cage. After rat SBP measurement, the blood sample was collected by puncture of the inferior vena cava. After centrifugation, the serum was collected. The serum urea and creatinine content and 24-h  urinary protein level were determined using an automatic biochemical analyzer (Hitachi 7600, Tokyo, Japan) [28].

### 2.5. Ultrastructure of Renal Tissue

Renal cortex tissue (2 × 2 × 2 mm^3^) was fixed with 3% glutaraldehyde solution and then prepared into sections for electron microscopy [28]. The ultrastructure of renal sections was observed under a JEM-1230 transmission electron microscope (JEOL, Tokyo, Japan).

### 2.6. *Ex Vivo* Incubation Experiments of Renal Tissue Slices

After the rats were anesthetized with 25% urethane, the abdominal cavity was quickly opened. The kidney was removed and stored in aerated sterile Krebs buffer (pH 7.4) on ice. The kidney cross-section was placed in a tissue microtome with 0.1% low-melting-point agarose in ice-cold phosphate-buffered saline (PBS) to prepare renal cortex slices (300 ± 50 *μ*m). The slices were incubated in continuously aerated (95% O_2_ : 5% CO_2_) Dulbecco's modified Eagle medium (DMEM)/F12 medium at 37°C for 3 h. Finally, the incubation medium and renal slices were collected and tested.

### 2.7. Inhibition of CBS Activity in Renal Slices

HA was used in the *ex vivo* incubation experiments to suppress renal CBS activity. The Dahl renal slices were divided into the NS (incubation medium with 137 mM NaCl) [34], HS, HS+Tau, and HS+Tau+HA groups. HS incubation medium contained 200 mM NaCl [34, 35]. The concentrations of taurine and HA in the incubation medium were 10 mM and 50 *μ*M, respectively [36, 37].

### 2.8. CBS Knockdown in Rat Renal Tissue

Lentivirus containing CBS-small hairpin RNA (shRNA) or scramble-shRNA was purchased from Cyagen Biosciences Inc. (Guangzhou, China). After four-week-old male Dahl rats (weighing about 150 g) were anesthetized, a small incision was made in the renal area on the back of the rats to expose the kidney. Each kidney was administrated with 100 *μ*L of scramble-shRNA or CBS-shRNA lentivirus (1 × 10^9^ TU/mL) through kidney puncture. The same procedure was used for both kidneys. After two weeks of lentivirus infection, the *ex vivo* incubation experiments were performed using the renal tissue slices. Dahl rats transfected with scramble-shRNA lentivirus were divided into three groups (*n* = 8 per group): the Dahl+NS+scramble-shRNA, Dahl+HS+scramble-shRNA, and Dahl+HS+Tau+scramble-shRNA groups. Dahl rats transfected with CBS-shRNA lentivirus were also divided into three groups (*n* = 8 per group): the Dahl+NS+CBS-shRNA, Dahl+HS+CBS-shRNA, and Dahl+HS+Tau+CBS-shRNA groups. The NS and HS incubation medium contained 137 mM and 200 mM NaCl, respectively. The incubation medium contained a taurine concentration of 10 mM.

### 2.9. Taurine Content Determination

Taurine content was determined by Beijing Mass Spectrometry Medical Research Co., Ltd (Beijing, China). The renal tissue was homogenized in deionized water on ice. The homogenate (50 *μ*L) was mixed with 50 *μ*L of protein precipitation agent and centrifuged at 13,200 rpm for 4 min. The supernatant (10 *μ*L) was mixed with 50 *μ*L labeling buffer and centrifuged for a few seconds. Derivatization solution (20 *μ*L) was added and centrifuged for a few seconds, then reacted for 15 min at 55°C. The above mixture (50 *μ*L) was analyzed using a high-performance liquid chromatographer (AB Company, Carlsbad, CA, USA). The liquid chromatography column was MSLAB 45+AA-C18 (150 × 4.6 mm, 5 *μ*m). The mobile phase included the water phase (water containing 1‰ formic acid) and organic phase (acrylonitrile containing 1‰ formic acid). The methanol elution gradient was 90% (0–1 min), 90–30% (1–12 min), 30–0% (12–12.1 min), 0% (12.1–15 min), 0–90% (15–15.1 min), and 90% (15.1–20 min). The taurine content in the renal tissue was determined in the positive ionization mode.

### 2.10. H_2_S Content Determination

H_2_S levels in serum and renal tissue were measured by the sensitive sulfur electrode method [37]. The renal tissue was homogenized with PBS and centrifuged, and the supernatant was collected to measure the protein and H_2_S concentrations. The front end (10 mm) of the polarized H_2_S electrode (WPI, Shanghai, China) was inserted into the sample at room temperature, and the current output (pA) increased. After reaching a plateau, the pA was recorded. A standard curve was drawn based on the concentration of sodium sulfide and pA. The H_2_S content in the sample was calculated based on this standard curve. The H_2_S content in the tissue homogenate was then corrected for protein concentration.

### 2.11. Real-Time Quantitative Polymerase Chain Reaction (RT-qPCR)

The rat renal tissues were ground into powder in liquid nitrogen, followed by the addition of TRIzol for homogenization and lysis. Chloroform was added to remove protein and DNA. RNA was selectively precipitated by isopropanol, washed with ethanol, dissolved in ribozyme-free water, and finally reverse-transcribed into cDNA [38]. RT-qPCR was carried out on an Applied Biosystems 7300 system (Carlsbad, CA, USA). Each reaction contained a mixture of 2 × mix, primers (7.5 *μ*M), TaqMan probe (5 *μ*M), cDNA, and purified water. The PCR program was 10 min at 95°C, followed by 40 cycles of 15 s at 95°C and 1 min at 60°C. Data were compared quantitatively with the relative CT value (2^-*ΔΔ*CT^). *β*-Actin was used as the internal reference.  Table 1 shows the primer and TaqMan probe sequences.

### 2.12. Western Blot

Renal protein was extracted using lysis buffer containing 50 mM Tris base, 150 mM NaCl, 1 mM EDTA, 6 mM sodium deoxycholate, 1% NP-40, protease inhibitor, phosphatase inhibitor, and phenylmethylsulfonyl fluoride (PMSF). After centrifugation, the supernatants were used for protein quantification and Western blot [39]. The proteins were separated by polyacrylamide electrophoresis and transferred to a nitrocellulose membrane, which was then blocked and incubated with antibodies. ECL luminescent solution was added to the membrane which was then placed in a gel imaging instrument (Gene Company Limited Company, Hong Kong, China) for exposure. Optical density values of protein bands were determined by AlphaEase FC software. The optical density of the corresponding internal reference *β*-actin or GAPDH was used for correction.

### 2.13. Detection of CBS, MPST, and CSE Activity in Renal Tissues

Renal homogenate was incubated with protein A/G at 4°C for 1 h and centrifuged at 8,000 g for 10 min. Supernatant was incubated with primary antibody against corresponding protein overnight. Protein A/G was then added, and the mixture was incubated for 4 h. After centrifugation, precipitated protein was eluted.

According to Stipanuk's method in the published literature [40], 10 *μ*g of protein was added to 1 mL of reaction solution containing 100 mM potassium phosphate buffer, 10 mM L-cysteine, and 2 mM pyridoxal 5′-phosphate and incubated at 37°C for 2 h. Renal H_2_S contents were measured using the free radical detection analyzer TBR4100. The rate of H_2_S production was calculated to reflect the CBS and CSE activity (nmol/h/*μ*g).

To detect MPST activity, 10 *μ*g of protein was added into 1 mL of reaction solution including PBS and 2 mM *β*-mercaptopyruvate and incubated for 2 h. H_2_S contents were measured using the free radical detection analyzer TBR4100, and the rate of H_2_S production was calculated to reflect the MPST activity (nmol/h/*μ*g) [41].

### 2.14. Determination of Renin, Angiotensin II (AngII), and Aldosterone Content by Enzyme-Linked Immunosorbent Assay (ELISA)

ELISA kits for renin, AngII, and aldosterone were purchased from Shanghai Bogu Biotechnology Co., Ltd (Shanghai, China). The concentrations of renin, AngII, and aldosterone in the rat renal tissue were determined by ELISA and standardized with total protein content according to the manufacturer's instructions.

### 2.15. Determination of Oxidative Stress Indicators in Renal Tissue

The renal tissues were homogenized in PBS and centrifuged at 10,000 g for 10 min to obtain supernatant. According to the manufacturer's instructions provided in the kits (Nanjing Jiancheng Bioengineering Institute, Nanjing, China), the biochemical colorimetry method [42] was used to determine the activities of superoxide dismutase (SOD), catalase (CAT), glutathione peroxidase (GSH-PX), and myeloperoxidase (MPO); the contents of oxidized glutathione (GSSG), reduced glutathione (GSH), malondialdehyde (MDA), hydrogen peroxide (H_2_O_2_), hydroxyl radical (^·^OH), and carbon monoxide (CO); and total antioxidant capacity (T-AOC).

### 2.16. Immunofluorescence Staining

The frozen sections of rat aorta were washed with PBS, blocked with 5% BSA, and then incubated with primary antibodies overnight at 4°C. The sections were then washed with PBS three times and incubated with secondary antibodies (Invitrogen, Carlsbad, CA, USA) for 1.5 h at room temperature away from light. After washing, sections were stained with DAPI (ZSGB-Bio, ZLI-9557). The fluorescence signals were captured under an Olympus confocal laser scanning microscope.

### 2.17. Statistical Analysis

Statistical analysis was performed using IBM SPSS 13.0 software. Data are expressed as the mean ± standard error (mean ± SE). Comparisons among multiple groups were performed using one-way analysis of variance (ANOVA) with Bonferroni's post hoc test. *P* values less than 0.05 are considered statistically significant.

## 3. Results

### 3.1. The Renal Endogenous Taurine Pathway Was Significantly Downregulated during Salt-Sensitive Hypertensive Renal Injury

**Figure 1:**
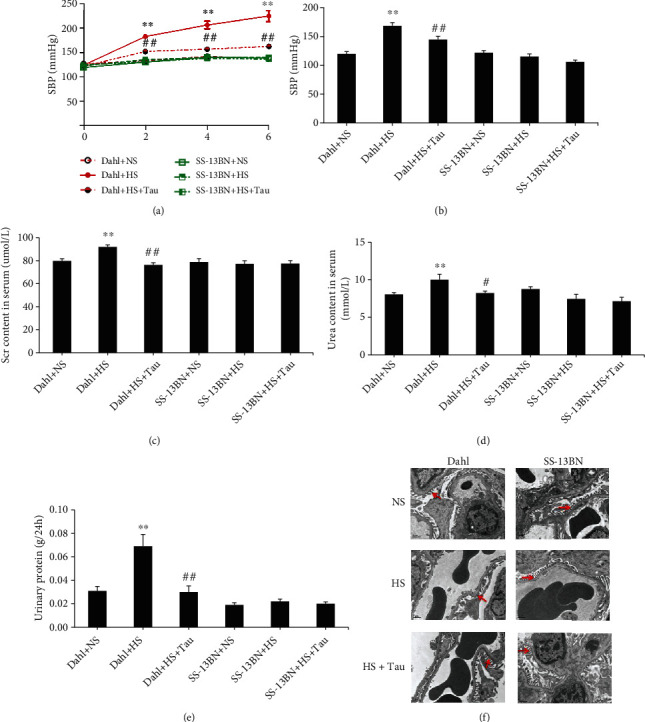
Taurine increment inhibited high-salt (HS) diet-induced renal damage in Dahl rats. (a) Changes of tail arterial systolic blood pressure (SBP) of Dahl and SS-13BN rats treated with normal salt (NS) diet, HS diet, or HS feeding supplemented with 2% taurine in drinking water (HS+Tau) for 2, 4, and 6 weeks, measured using an intelligent noninvasive blood pressure monitor. (b) Changes in SBP after 6 weeks of HS diet feeding measured by the carotid artery catheter method. (c–e) Changes of serum creatinine (Scr) content (c), serum urea content (d), and the 24 h urinary protein level (e) in rats. (f) Electron microscope examination of rat renal ultrastructure (transmission electron microscopy, magnification: 8000x). → indicates foot process of glomerular podocytes. *n* = 10 for each group. ^∗∗^*P* < 0.01 vs. Dahl+NS; ^#^*P* < 0.05 and ^##^*P* < 0.01 vs. Dahl+HS.

**Figure 2:**
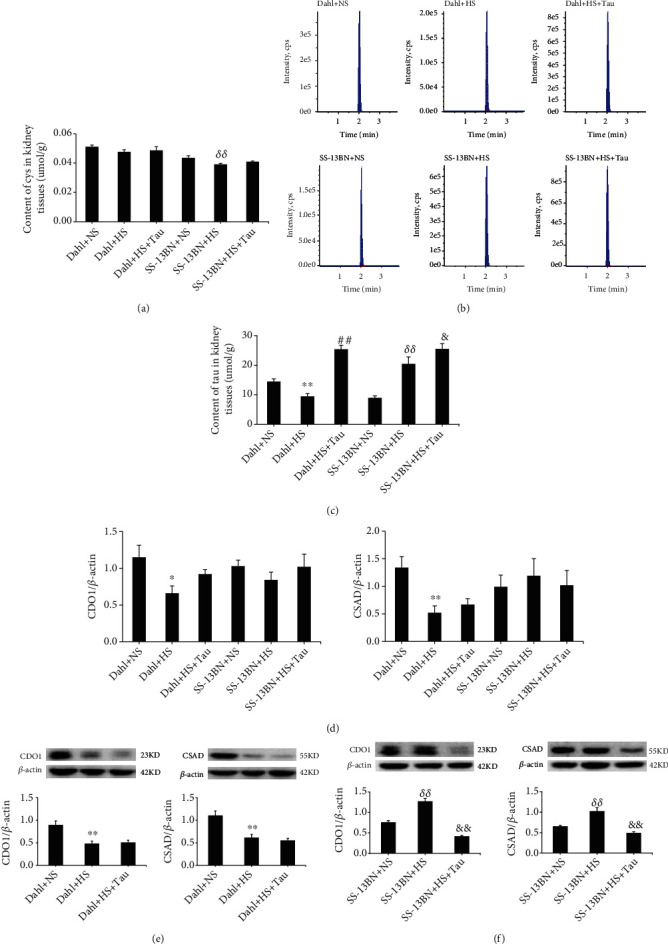
High-salt (HS) diet downregulated renal endogenous taurine-generating pathway in Dahl rats. (a) Changes of cysteine (Cys) contents in rat renal tissues. (b) The representative high-performance liquid chromatograms of taurine (Tau) in rat renal tissues. (c) Changes in Tau contents in rat renal tissues. (d) CDO1 and CSAD mRNA levels in rat renal tissues. (e) Protein levels and quantification of CDO1 and CSAD in the kidneys of Dahl rats. (f) Protein levels and quantification of CDO1 and CSAD in the kidneys of SS-13BN rats. *n* = 10 for each group. ^∗^*P* < 0.05 and ^∗∗^*P* < 0.01 vs. Dahl+normal salt (NS); ^##^*P* < 0.01 vs. Dahl+HS; *^*δδ*^P* < 0.01 vs. SS-13BN+NS; ^&^*P* < 0.05 and ^&&^*P* < 0.01 vs. SS-13BN+HS.

To examine the role of the endogenous taurine pathway in salt-sensitive hypertensive renal damage, both salt-sensitive Dahl rats and salt-resistant SS-13BN rats were given NS and HS diets. Compared with the Dahl+NS group, the tail arterial SBP of the Dahl+HS group was consistently and markedly increased after 2, 4, and 6 weeks of HS feeding according to the tail-cuff method (Figure 1(a)). The results of the carotid artery catheterization method also revealed a significant increase in arterial SBP after 6 weeks of HS feeding (Figure 1(b)). Serum contents of creatinine and urea and the 24-h urinary protein level were elevated in HS Dahl rats (Figures 1(c)–1(e)). The results of electron microscopy showed that glomerular podocytes were fused in the Dahl+HS group (Figure 1(f)). These data demonstrate that the salt-sensitive hypertensive renal injury rat model was successfully established. Interestingly, the taurine content and mRNA and protein levels of CDO1 and CSAD were significantly downregulated in the kidneys of HS-fed Dahl rats compared with NS-fed rats (Figures 2(b)–2(e)), while the renal cysteine content remained unchanged (Figure 2(a)).

However, the taurine content and CDO1 and CSAD protein levels were higher in the kidneys of SS-13BN+HS rats than those in SS-13BN+NS rats (Figures 2(b), 2(c), and 2(f)), while the CDO1 and CSAD mRNA expression in the renal tissues did not differ between the two groups (Figure 2(d)). Furthermore, the renal cysteine content decreased in the SS-13BN+HS group (Figure 2(a)). HS diet did not affect the blood pressure and renal function of SS-13BN rats (Figure 1). These results suggest that the HS diet downregulated the endogenous taurine pathway and impaired renal function in salt-sensitive hypertensive Dahl rats.

### 3.2. Downregulation of Renal Taurine Promoted Renal Damage in Salt-Sensitive Hypertension

To investigate the functional significance of renal taurine downregulation during salt-sensitive hypertensive renal injury, the HS diet of both the SS-13BN rats and Dahl rats was supplemented with or without 2% taurine in drinking water (HS+Tau or HS). The renal taurine content was increased in the Dahl+HS+Tau group compared with the Dahl+HS group (Figures 2(b) and 2(c)). This upregulation of the renal taurine contents was associated with a marked decrease in the SBP (Figures 1(a) and 1(b)), serum creatinine content (Figure 1(c)), serum urea content (Figure 1(d)), and 24 h urinary protein level (Figure 1(e)). Electron microscopy results showed that the fusion of glomerular podocytes was significantly reduced in the Dahl+HS+Tau group compared with the Dahl+HS group (Figure 1(f)). Taurine supplementation in SS-13BN rats given the HS diet increased the taurine content in the renal tissues (Figures 2(b) and 2(c)) and decreased CDO1 and CSAD protein expression (Figure 2(f)); however, it did not change the CDO and CSAD mRNA expression (Figure 2(d)), the SBP level of rats, or the function and structure of the kidney compared to the HS-fed SS-13BN rats without taurine treatment (Figure 1). These data suggest that exogenous taurine supplementation restored the renal taurine of Dahl rats to normal levels, which could antagonize HS-induced hypertensive renal damage. These findings imply that high salt downregulated the endogenous taurine pathway to cause renal injury during salt-sensitive hypertension.

### 3.3. Endogenous Taurine Reduction Suppressed the Renal CBS/H_2_S Pathway in Rats with Salt-Sensitive Hypertensive Renal Damage

**Figure 3:**
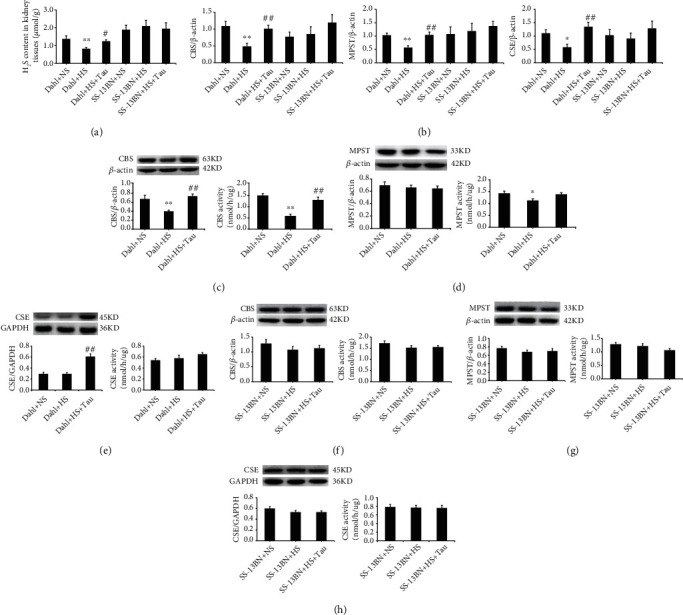
Taurine downregulation induced by high-salt (HS) diet reduced the cystathionine-*β*-synthase (CBS)/hydrogen sulfide (H_2_S) pathway in Dahl kidneys. (a) H_2_S content in rat renal tissues. (b) Renal mRNA levels of CBS, mercaptopyruvate sulfurtransferase (MPST), and cystathionine-*γ*-lyase (CSE). (c–e) Protein expression and activity of CBS (c), MPST (d), and CSE (e) in Dahl rat kidneys. (f–h) Protein expression and activity of CBS (f), MPST (g), and CSE (h) in SS-13BN rat kidneys. *n* = 10 for each group. ^∗^*P* < 0.05 and ^∗∗^*P* < 0.01 vs. Dahl+normal salt (NS); ^#^*P* < 0.05 and ^##^*P* < 0.01 vs. Dahl+HS.

Taurine treatment is known to increase plasma H_2_S levels in prehypertensive patients [15], and previous studies have shown the possible renoprotective effect of H_2_S [28]. Therefore, the changes in the endogenous H_2_S-producing pathway were analyzed to aid in understanding the mechanism by which downregulated taurine contributed to renal injury in HS diet-fed Dahl rats. HS diet-fed Dahl rats exhibited a significant decrease in renal H_2_S content (Figure 3(a)), CBS mRNA, protein levels, and activity, MPST mRNA levels and activity, and renal CSE mRNA levels compared with those fed an NS diet (Figures 3(b)–3(d)). Meanwhile, MPST protein expression and CSE protein expression and activity in the renal tissues were not significantly different between the Dahl+NS and Dahl+HS groups (Figures 3(d) and 3(e)). Notably, renal H_2_S content (Figure 3(a)), CBS mRNA, protein expression, and activity (Figures 3(b) and 3(c)), MPST mRNA levels (Figure 3(b)), and CSE mRNA and protein levels (Figures 3(b) and 3(e)) in renal tissues were markedly elevated by taurine supplementation in HS-induced Dahl rats; meanwhile, MPST activity and protein expression (Figure 3(d)) and CSE activity (Figure 3(e)) did not change.

There were no differences in the renal H_2_S content, CBS mRNA and protein expression, and CBS, MPST, and CSE activity among the SS-13BN+NS, SS-13BN+HS, and SS-13BN+HS+Tau groups (Figures 3(a), 3(b), and 3(f)–3(h)). These data suggest that taurine supplementation upregulated the HS diet-inhibited renal CBS/H_2_S levels in Dahl rats. Furthermore, the decrease in taurine caused by the HS diet suppressed the renal H_2_S-generating pathway during salt-sensitive hypertensive renal damage.

### 3.4. Taurine Downregulation Facilitated Renal Oxidative Stress in Salt-Sensitive Hypertensive Rats

**Figure 4:**
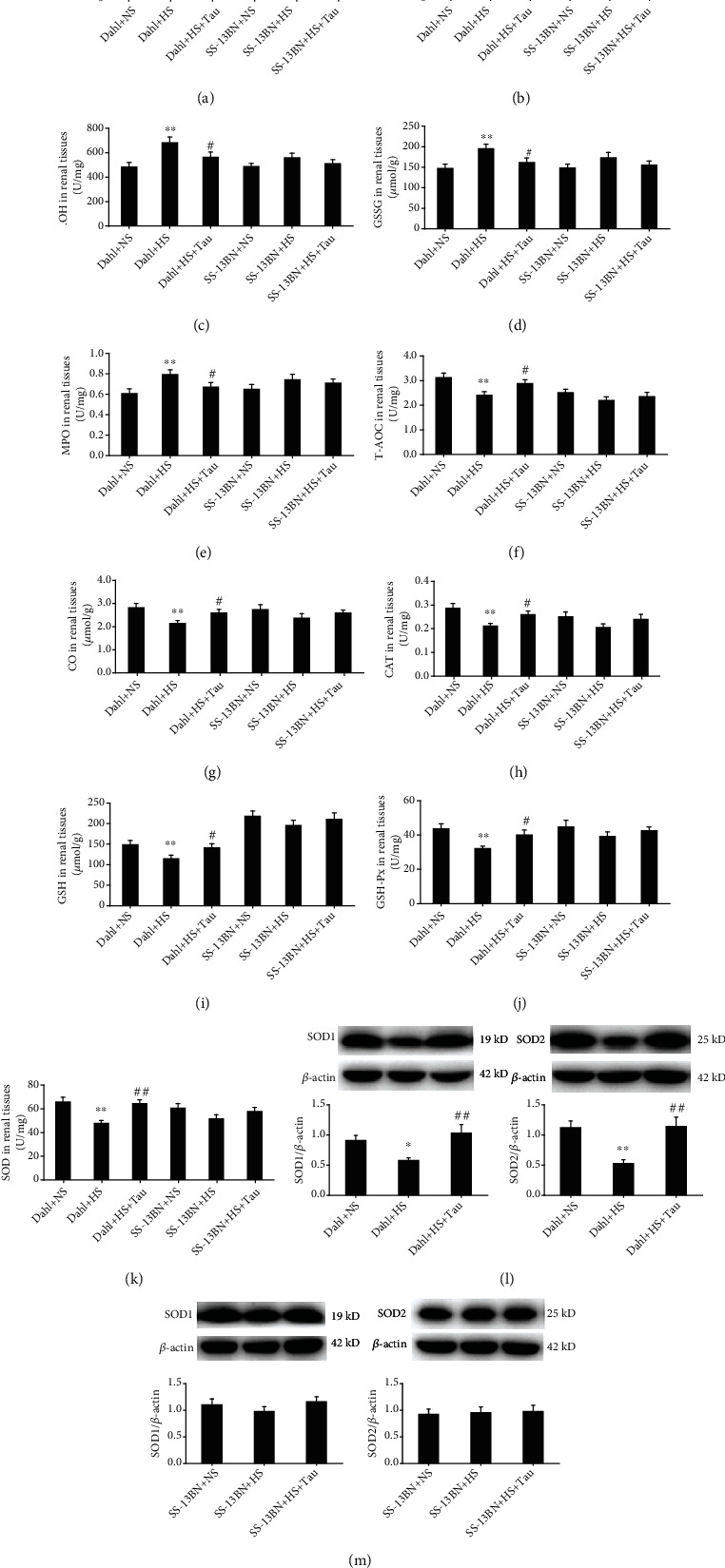
High-salt (HS) diet-caused decrease in taurine promoted oxidative stress and impaired antioxidative capacity in Dahl rat kidneys. (a–d) Malondialdehyde (MDA) (a), H_2_O_2_ (b), ^·^OH (c), and oxidized glutathione (GSSG) contents (d) in rat renal tissues. (e) Myeloperoxidase (MPO) activity in rat renal tissues. (f) Total antioxidant capacity (T-AOC) in renal tissues. (g) Carbon monoxide (CO) content in rat renal tissues. (h) Catalase (CAT) activity in rat renal tissues. (i) Reduced glutathione (GSH) content. (j) Glutathione peroxidase (GSH-PX) activity. (k) Superoxide dismutase (SOD) activity. (l) SOD1/2 protein levels in Dahl kidneys. (m) SOD1/2 protein levels in SS-13BN kidneys. *n* = 10 for each group. ^∗^*P* < 0.05 and ^∗∗^*P* < 0.01 vs. Dahl+normal salt (NS); ^#^*P* < 0.05 and ^##^*P* < 0.01 vs. Dahl+HS.

Oxidative stress is crucial in the pathogenesis of renal damage. Therefore, the impact of taurine on various oxidative damage substances and antioxidant substances in the rat renal tissues was examined. The renal contents of MDA, H_2_O_2_, ^·^OH, and GSSG and the MPO activity were higher in Dahl rats fed an HS diet than those fed an NS diet (Figures 4(a)–4(e)); meanwhile, the T-AOC (Figure 4(f)), CO, and GSH contents (Figures 4(g) and 4(i)), CAT, GSH-PX, and SOD activities (Figures 4(h), 4(j), and 4(k)), and SOD1 and SOD2 protein expression (Figure 4(l)) were lower in the kidneys of HS diet-fed Dahl rats than in those fed an NS diet. More importantly, oxidative damage index levels significantly decreased (Figures 4(a)–4(e)), and antioxidant index levels significantly increased (Figures 4(f)–4(k)) in the kidneys of Dahl+HS+Tau group rats.

However, the renal contents of MDA, H_2_O_2_, ^·^OH, GSSG, T-AOC, CO, and GSH (Figures 4(a)–4(d), 4(f), 4(g), and 4(i)), the activities of MPO, CAT, GSH-PX, and SOD (Figures 4(e), 4(h), 4(j), and 4(k)), and the SOD1 and SOD2 protein levels (Figure 4(m)) did not differ among the SS-13BN+NS, SS-13BN+HS, and SS-13BN+HS+Tau groups. These data indicated that taurine supplementation inhibited oxidative stress and promoted antioxidant capacity in the kidneys of HS diet-fed Dahl rats. Furthermore, the decrease in taurine caused by the HS diet promoted renal oxidative stress.

### 3.5. Taurine Downregulation Enhanced Renal RAAS Activation in Salt-Sensitive Hypertensive Rats

**Figure 5:**
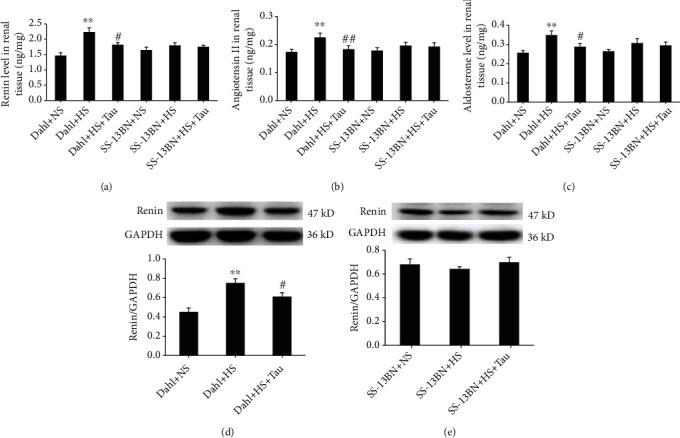
Taurine reduction by the high-salt (HS) diet activated the renin-angiotensin-aldosterone system (RAAS) in Dahl rat kidneys. (a–c) Renal contents of renin (a), angiotensin II (b), and aldosterone (c). (d, e) Protein levels of renin in Dahl rat kidneys (d) and SS-13BN rat kidneys (e). *n* = 10 for each group. ^∗∗^*P* < 0.01 vs. Dahl+normal salt (NS); ^#^*P* < 0.05 and ^##^*P* < 0.01 vs. Dahl+HS.

The RAAS is also known to be activated by high salt and acts as another important factor in the pathogenesis of salt-sensitive hypertensive renal damage. As such, we investigated the influence of taurine on renal RAAS activation in HS diet-fed Dahl rats. High salt activated renal RAAS in Dahl rats, and renin, AngII, and aldosterone contents and renin protein expression were increased (Figures 5(a)–5(d)). Meanwhile, the administration of taurine to HS diet-fed Dahl rats significantly reduced renal contents of renin, AngII, and aldosterone and renin protein expression (Figures 5(a)–5(d)).

In SS-13BN rats, the renal contents of renin, AngII, and aldosterone and renin protein expression were not markedly different among the NS, HS, and HS+Tau groups (Figures 5(a)–5(c) and 5(e)). These data suggest that the decreased taurine caused by the HS diet promoted renal RAAS activation in Dahl rats.

### 3.6. Endogenous Taurine Reduction Caused by High Salt Contributed to Renal Injury through CBS/H_2_S Pathway Inhibition in Dahl Rats

**Figure 6:**
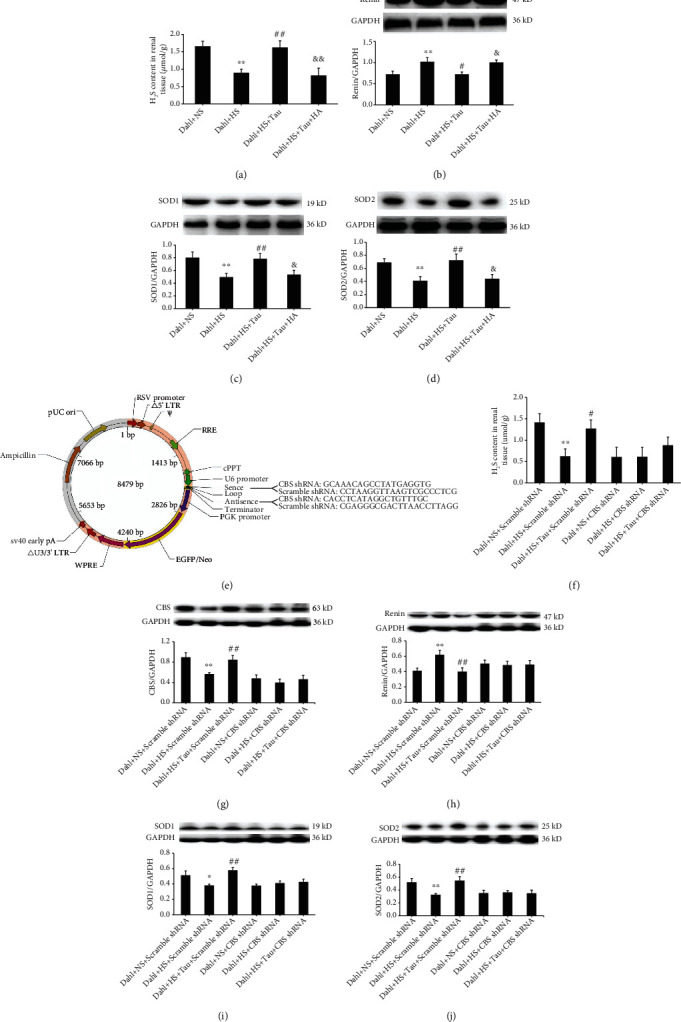
Inhibition of cystathionine-*β*-synthase (CBS) activity or knockdown of CBS expression abolished the action of high-salt treatment or taurine increment on the RAAS and oxidative stress in Dahl rat renal slices *ex vivo*. (a–d) H_2_S content (a) and renin (b), SOD1 (c), and SOD2 (d) levels in the renal slices of Dahl rats incubated with medium containing normal salt (NS, 137 mM), high salt (HS, 200 mM), high salt (200 mM) plus taurine (10 mM) (HS+Tau), or high salt (200 mM) plus taurine (10 mM) and HA (50 *μ*M) (HS+Tau+HA). (e) The original sequence of scramble-shRNA and target sequence of CBS-shRNA and lentivirus vector map. (f–j) H_2_S content (f) and protein expressions of CBS (g), renin (h), SOD1 (i), and SOD2 (j) in the renal slices of Dahl rats transfected with scramble-shRNA or CBS-shRNA lentivirus and incubated with medium containing NS (137 mM), HS (200 mM), or HS (200 mM)+Tau (10 mM). *n* = 8 for each group. ^∗^*P* < 0.05 and ^∗∗^*P* < 0.01 vs. Dahl+NS; ^#^*P* < 0.01 and ^##^*P* < 0.01 vs. Dahl+HS; ^&^*P* < 0.05 and ^&&^*P* < 0.01 vs. Dahl+HS+Tau.

To explore whether taurine downregulation promoted renal injury through the endogenous H_2_S pathway, the Dahl renal slices were treated with HA, an inhibitor of CBS activity, in *ex vivo* experiments. The renal slices with HS medium incubation exhibited a decrease in H_2_S content (Figure 6(a)) and SOD1 and SOD2 protein expression (Figures 6(c) and 6(d)), but an increase in renin expression (Figure 6(b)). Meanwhile, supplementation of HS-treated renal slices with taurine upregulated H_2_S content (Figure 6(a)) and SOD1 and SOD2 protein expression (Figures 6(c) and 6(d)) but downregulated renin protein expression (Figure 6(b)). However, treatment with HA blocked taurine-induced H_2_S production, the inhibition of renin protein expression, and taurine-facilitated SOD1 and SOD2 levels in the Dahl renal slices incubated with HS (Figures 6(a)–6(d)).

To further confirm whether CBS mediated the effects of taurine on renal RAAS activation and oxidative stress, the kidneys of Dahl rats were transfected with CBS-shRNA lentivirus to knock down renal CBS (Figure 6(e)). Scramble-shRNA was used as a control. The Dahl renal slices were treated with normal medium, HS medium, and HS medium containing taurine after transfection with each lentivirus. The H_2_S content (Figure 6(f)) and CBS (Figure 6(g)), SOD1 (Figure 6(i)), and SOD2 (Figure 6(j)) protein expression were decreased in the renal slices of Dahl rats transfected with scramble-shRNA and incubated with HS medium, but renin protein expression was increased (Figure 6(h)). Meanwhile, taurine administration rescued the rats from this effect (Figures 6(f)–6(j)). The renal H_2_S content and CBS protein expression were significantly downregulated in the Dahl rats whose kidneys were transfected with CBS-shRNA lentivirus compared with those whose kidneys were transfected with scramble-shRNA lentivirus (Figures 6(f) and 6(g)). Notably, the H_2_S content and CBS, renin, SOD1, and SOD2 levels in Dahl rat renal slices transfected with CBS-shRNA were not affected by HS treatment or HS and taurine treatment (Figures 6(f)–6(j)). These data suggested that downregulation of endogenous taurine caused by the HS treatment promoted RAAS activation and oxidative stress in the kidneys of Dahl rats by inhibiting the renal CBS/H_2_S pathway.

### 3.7. High-Salt Diet Caused a Reduction in Endogenous Taurine in association with Endothelial Dysfunction in Dahl Rats

**Figure 7:**
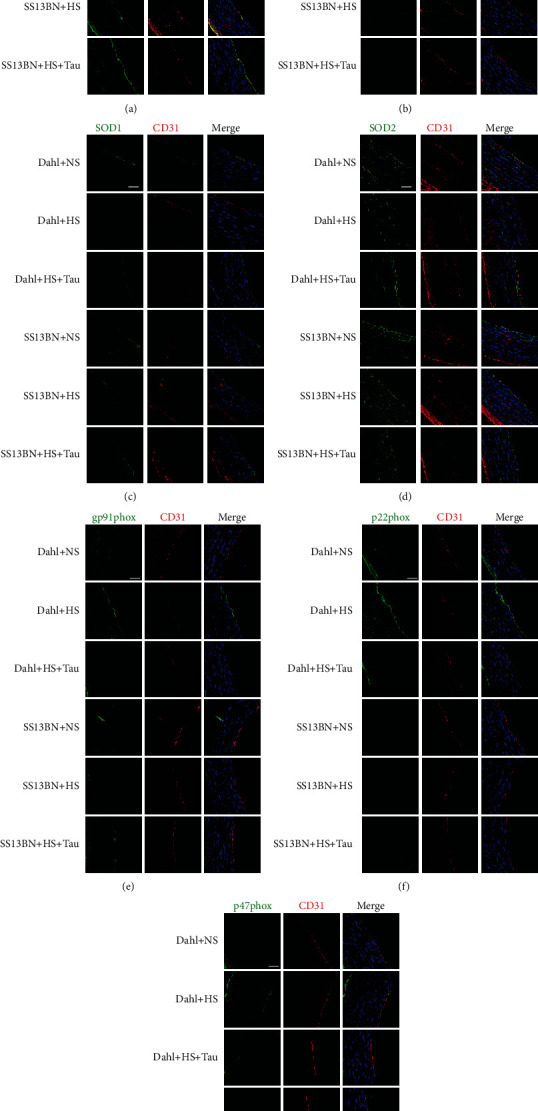
Endogenous taurine reduction by high-salt (HS) diet in Dahl rats is associated with endothelial dysfunction. (a–d) Immunofluorescence detection of protein expressions (green fluorescence) of CBS (a), renin (b), SOD1 (c), SOD2 (d), gp91phox (e), p22phox (f), and p47phox (g) in rat aortic endothelial cells. Red fluorescence represents CD31, a marker for endothelial cells. Blue fluorescence represents the nuclei. Scale bars: 40 *μ*m.

Considering that taurine could improve the repair of damaged endothelial function in SHRs through its antioxidant effect [43], we investigated the contribution of taurine downregulation by the HS diet to endothelial dysfunction. The results showed that the HS diet downregulated CBS, SOD1, and SOD2 protein expression but upregulated renin, gp91phox, p22phox, and p47phox protein expression in the aortic endothelial cells of Dahl rats. Meanwhile, taurine supplementation increased CBS, SOD1, and SOD2 expression but decreased renin, gp91phox, p22phox, and p47phox levels in the aortic endothelial cells of HS diet-fed Dahl rats (Figure 7). There were no differences in these indicators in aortic endothelial cells among the SS-13BN+NS, SS-13BN+HS, and SS-13BN+HS+Tau groups (Figure 7). These data suggest that taurine downregulation caused by the HS diet is associated with endothelial dysfunction.

## 4. Discussion

**Figure 8:**
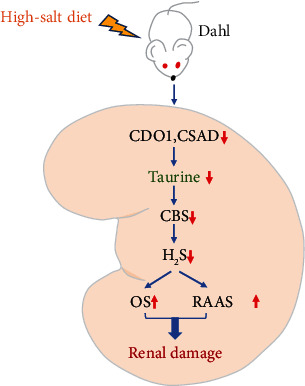
A schematic diagram. Downregulation of the endogenous taurine pathway is an important mechanism for renal injury during salt-sensitive hypertension. High salt downregulated endogenous taurine production to reduce renal cystathionine-*β*-synthase (CBS) expression and activity, subsequently decreasing renal H_2_S generation, facilitating renin-angiotensin-aldosterone system (RAAS) activation and oxidative stress, and ultimately leading to renal damage in salt-sensitive Dahl rats.

In the present study, we first revealed a novel mechanism for salt-sensitive hypertensive renal injury. This mechanism shows that downregulation of the endogenous taurine pathway is a crucial mechanism for renal injury in salt-sensitive hypertension. Furthermore, our study clarified that the decrease in taurine caused by an HS diet suppresses endogenous H_2_S production by downregulating renal CBS expression and activity. This process subsequently promotes the activation of the RAAS and oxidative stress in renal tissue, thereby leading to renal damage in Dahl rats (Figure 8).

Taurine can be produced by CDO oxidation and CSAD decarboxylation in organisms with cysteine as the substrate [8]. Taurine exerts a variety of biological effects, including the inhibition of RAAS activity and oxidative stress, vasorelaxation, and the facilitation of water and sodium metabolism [9, 10, 44]. Taurine is closely related to the occurrence of HS-induced hypertension. Taurine can lower blood pressure, reduce the levels of vasoconstrictor active substances, and upregulate vasodilator substance content in Wistar rats with hypertension induced by abdominal aortic stenosis and HS [45]; these results suggest that taurine inhibits the occurrence of renal hypertension possibly through regulating the levels of vasoactive substances. Ideishi et al. later found that exogenous taurine supplementation inhibited the development of hypertension and protected against heart damage associated with increased kallikrein in salt-sensitive Dahl rats [46]. In the stroke-prone SHRs fed an HS diet, taurine supplementation significantly reduced blood pressure and alleviated ventricular hypertrophy [47]. Furthermore, the administration of taurine inhibited LOX-1 expression and exerted an antioxidant effect in the renal tissue of Dahl rats [48]. These studies suggest that exogenous taurine supplementation could prevent the development of hypertension and might have a possible protective effect on hypertension-related target organ damage. However, it is currently unclear how sulfur-containing amino acid metabolism-produced taurine is involved in the mechanism underlying salt-sensitive hypertensive renal damage.

To address this gap, Dahl rats were herein administrated with an HS diet as an animal model of hypertensive renal injury. Salt-resistant SS-13BN rats were used as experimental controls. First, the changes in endogenous taurine and its key enzymes were detected. The results showed that the blood pressure of Dahl rats continued to rise after HS diet administration and increased dramatically at 6 weeks, accompanied by impaired renal function and structure. Meanwhile, blood pressure was stable, and renal function and structure were normal in SS-13BN rats fed with an HS diet. These results confirmed that the salt-sensitive hypertensive renal injury model was successfully established. In this model, we found that the endogenous taurine content in the renal tissue was significantly reduced and the mRNA and protein expressions of the taurine-producing enzymes, CDO1 and CSAD, were downregulated. Meanwhile, the level of cysteine, the substrate for taurine synthesis, remained unchanged. The HS diet upregulated taurine content and CDO1 and CSAD protein expression but downregulated cysteine levels in the renal tissue of SS-13BN rats. These results indicate that endogenous taurine and its key enzymes were significantly downregulated during salt-sensitive hypertensive renal damage.

To further explore the significance of endogenous taurine downregulation in renal injury, we supplemented taurine to HS diet-fed Dahl rats from the perspective of “gain-of-function.” Results showed that the taurine content in the HS-administrated Dahl renal tissue was increased after exogenous taurine supplementation, rat SBP was reduced, and the renal functional and structural damage were alleviated. These results suggest that downregulation of the endogenous taurine pathway is an important part of the pathogenesis of salt-sensitive hypertensive renal damage.

The mechanism by which the downregulation of endogenous taurine leads to salt-sensitive hypertensive renal injury is currently unclear. A previous study showed that the plasma H_2_S level was increased after taurine supplementation in SHRs [15]. The H_2_S donor NaHS increased renin protein expression in As4.1 cells [49], and CSE overexpression promoted H_2_S production and inhibited isoproterenol-induced renin release [49]. The serum levels of ACE, AngI, and AngII were significantly increased in L-NAME-induced hypertensive rats, while exogenous taurine supplementation reduced them; these results indicate that the RAAS might mediate the antihypertensive effect of taurine [11]. Chiba et al. [48] found that taurine inhibited LOX-1 expression to protect against salt-sensitive hypertension, and the kidney is an important organ for maintaining blood pressure. These results suggest that oxidative stress might be involved in the mechanism by which taurine regulates salt-sensitive hypertension. Our group previously found that H_2_S inhibited superoxide anion generation in human umbilical vein endothelial cells exposed to high salt [34], indicating that H_2_S could inhibit oxidative stress stimulated by high salt in endothelial cells. It was also found that H_2_S reduced renin, AngII, and aldosterone contents, decreased the generation of free oxygen radicals, and enhanced antioxidant enzyme activity and expression in the renal tissue of HS diet-fed Dahl rats [24, 28]. Therefore, we hypothesized that the decreased endogenous taurine might reduce endogenous H_2_S production in the kidney to promote renal RAAS activation and oxidative stress damage, thereby leading to salt-sensitive hypertensive renal damage. Thus, we conducted the following research to examine whether endogenous taurine downregulation contributed to renal damage in salt-sensitive hypertension and its possible mechanisms.

This study suggested that an HS diet reduced renal taurine levels and increased the renin, AngII, and aldosterone contents and renin protein levels in Dahl rat renal tissue; meanwhile, exogenous taurine supplementation increases taurine levels in the kidney significantly inhibiting RAAS activation. However, the HS diet did not activate the RAAS in the renal tissue of SS-13BN rats, which exhibited an increase in renal taurine content. Furthermore, the HS diet promoted renal oxidative stress, while an increase in taurine levels inhibited this oxidative stress in Dahl rats. The MDA, H_2_O_2_, ^·^OH, and GSSG contents and MPO activity reflect the degree of oxidative stress damage [50]; meanwhile, T-AOC and the CO, GSH, CAT, GSH-PX, SOD1, and SOD2 contents represent antioxidant capacity [51]. This study showed that supplementing HS diet-fed Dahl rats with taurine significantly reduced the renal MDA, H_2_O_2_, ^·^OH, GSSG, CO, and GSH contents and MPO activity but enhanced renal T-AOC, CAT, GSH-PX, and SOD activity. However, the HS diet could not induce oxidative stress in SS-13BN renal tissue with increased taurine levels. These results demonstrated that the downregulation of taurine caused by the HS diet promoted renal RAAS activation and oxidative stress in Dahl rats.

Interestingly, this study indicated that endogenous taurine reduction inhibited the renal H_2_S-generating pathway in salt-sensitive hypertensive Dahl rats. CBS, CSE, and MPST, which catalyze endogenous H_2_S production, are expressed in the kidney [18]. This study revealed that an HS diet downregulated H_2_S levels, CBS expression and activity, MPST mRNA levels and activity, and CSE mRNA levels in the renal tissue of Dahl rats, while taurine increment restored the renal H_2_S-generating pathway. The renal endogenous H_2_S pathway was not altered in the HS or HS+Tau groups of SS-13BN rats.

To determine whether a decrease in the renal H_2_S pathway could mediate the contribution of endogenous taurine reduction to renal damage, Dahl rat renal slices were treated with the CBS activity inhibitor HA in the *ex vivo* experiments. The results showed that HA treatment could significantly reduce renal H_2_S content, block the suppressive effect of taurine on renin protein levels, and abolish the enhancement effect of taurine on SOD1 and SOD2 contents in the presence of high salt. These data implied that taurine reduction by high salt promoted the activation of the RAAS and oxidative stress in Dahl renal tissue by inhibiting CBS activity.

Furthermore, Dahl rat kidneys were transfected with CBS-shRNA by using renal puncture to inhibit CBS expression. The kidneys were then isolated and cut into slices for *ex vivo* experiments. Taurine inhibited renin protein expression and promoted SOD1 and SOD2 levels in the scramble-shRNA-transfected Dahl rat kidneys incubated with high salt, which was abolished in renal slices with CBS knockdown. These results suggest that the endogenous CBS/H_2_S pathway mediated the action of taurine on RAAS activation and oxidative stress in Dahl rat renal slices exposed to high salt.

The concentration of NaCl in cell buffers such as PBS is 137 mM, and the control group in previous literature was mostly incubated with 137 mM NaCl [24, 34, 52]. Therefore, the NS group in this study was incubated with 137 mmol/L NaCl. Clinical studies have reported that hypernatremia patients developed renal impairment when the serum sodium concentration was close to 200 mM [53]. Many studies also used 200 mM NaCl as a high salinity stimulus [24, 34, 35, 54–56]. Moreover, our previous studies showed that 200 mM NaCl treatment could reduce the level of H_2_S in endothelial cells and kidney tissues [24, 34]. Therefore, the HS group in this study was treated with 200 mM NaCl. In a variety of isolated tissue and cell experiments including isolated rat heart, canine kidney cells, vascular smooth muscle cells, rat pancreatic islet cells, retinal glial cells, and Müller cells, 10 mM is the most common dose of taurine used to exert its effects [36, 57–64]. Our previous study has shown that 50 *μ*M HA could significantly inhibit H_2_S contents [37]; thus, a dose of 50 *μ*M HA was used in this study.

Trachtman et al. [65] found that 1% taurine in drinking water decreased blood pressure and reduced kidney and heart damage in SHRs; furthermore, its antihypertensive mechanism was independent of catecholamine, indicating that taurine might reduce blood pressure in SHRs by interacting with hormone systems involved in blood pressure regulation. Adedara et al. [66] reported that oral taurine (100 and 200 mg/kg) could significantly reduce blood pressure and ameliorate renal histological damage in L-NAME-induced hypertensive rats. The mechanism was related to the improvement of thyroid system function, an increase in nitric oxide levels, a decrease in myeloperoxidase activity, the enhancement of renal antioxidant enzyme activity, and the inhibition of renal oxidative stress. It was suggested that the evaluation of nitric oxide and the RAAS in the future would help to further explain the possible renal protective mechanism for taurine in L-NAME-induced hypertensive rats. In the present study, we revealed that endogenous taurine inhibited RAAS activation and oxidative stress through increasing the CBS/H_2_S pathway in renal tissue, ultimately alleviating renal injury and reducing blood pressure in salt-sensitive hypertensive rats. Previous studies reported that the downregulation of the endogenous H_2_S pathway was also an important factor in the pathogenesis of spontaneous hypertension [23] and L-NAME-induced hypertension [25]. These findings indicate that H_2_S pathway upregulation might also participate in the renal protection mechanism for taurine in these two models of hypertension; however, this mechanism still requires further study.

Previous studies have reported that 2% taurine in drinking water could significantly decrease blood pressure in L-NAME-induced hypertensive rats [11] and high fructose-induced hypertensive rats [67], upregulate CBS and CSE expression in the aorta of SHRs [15], alleviate kidney damage in hyperuricemia rats [68], and inhibit oxidative stress in the kidneys of diabetic rats [69]. Therefore, a 2% taurine supplementation scheme was chosen for this study. Previous studies have reported that 1% taurine in drinking water had only slight antihypertensive effects on Dahl rats fed with an HS (8%) diet [48], while 3% taurine in drinking water prevented hypertension in Dahl rats fed with an HS (4%) diet [46]. These two taurine supplementation schemes could reduce urinary protein [46, 48] and have antioxidant effects [11, 43, 70], indicating that they have renal protective effects.

Hypertensive chronic renal failure is a huge clinical burden. Lowering blood pressure is an important and widely used method to delay the progression of chronic kidney disease. Currently, there is almost no effective cure or prevention strategy for chronic kidney disease. Due to the lack of symptoms in the early stage of the disease, timely treatment is very challenging. An interesting clinical trial has confirmed that the change in blood pressure was negatively correlated with plasma taurine levels in prehypertensive patients treated with taurine [15]. Our present study suggested that taurine levels in the kidney tissue of salt-sensitive hypertensive rats were significantly reduced, which is an important factor in the pathogenesis of kidney injury. Therefore, taurine may be used as a new biomarker to predict the patient prognosis. Since taurine is an endogenous semiessential amino acid, the side effects of taurine treatment are expected to be small. As such, taurine supplementation may be a promising strategy for the treatment of hypertension and chronic kidney disease.

## 5. Conclusions

In summary, this study demonstrates that the downregulation of endogenous taurine in the renal tissue of Dahl rats is the main mechanism for salt-sensitive hypertensive renal damage. The decrease in endogenous taurine caused by an HS diet could reduce renal H_2_S production via the inhibition of CBS activity and the expression to promote RAAS activation and oxidative stress damage in the kidney, thereby resulting in renal damage in salt-sensitive Dahl rats. Our findings not only deepen the knowledge of the mechanism underlying salt-sensitive hypertensive renal injury but also provide new research ideas and potential therapeutic targets for renal injury. In the future, taurine-based drugs or foods will be expected to be developed for use in the treatment of renal damage.

## Figures and Tables

**Table 1:** Sequence of primer and TaqMan probe for rat target genes and *β*-actin.

Target		Sequence (5′-3′)	Product
CSAD	Forward	CTGGAGTGGCGCATCGA	80 bp
	Reverse	AACTCAAATCCTTCCCGCTTTT	
	TaqMan	CAGGCCTTTGCTCTCACTCGGTACTTGG	
CDO1	Forward	GGGAAAATCAGTGTGCCTACATT	121 bp
	Reverse	GCATGGCATGTATCGAAAGGT	
	TaqMan	TTACATCGAGTAGAGAACGTCAGCCACACAGAG	
CBS	Forward	CTCCGGGAGAAGGGTTTTGA	81 bp
	Reverse	CATGTTCCCGAGAGTCACCAT	
	TaqMan	AGGCACCTGTGGTCAACGAGTCTGG	
CSE	Forward	GCTGAGAGCCTGGGAGGATA	92 bp
	Reverse	TCACTGATCCCGAGGGTAGCT	
	TaqMan	CTGAGCTTCCAGCAATCATGACCCATG	
MPST	Forward	CGGCGCTTCCAGGTAGTG	131 bp
	Reverse	CTGGTCAGGAATTCAGTGAATGG	
	TaqMan	CCGCGCAGCTGGCCGTTT	
*β*-Actin	Forward	ACCCGCGAGTACAACCTTCTT	80 bp
	Reverse	TATCGTCATCCATGGCGAACT	
	TaqMan	CCTCCGTCGCCGGTCCACAC	

Note: the 5′ end of the TaqMan probe is labeled with FAM, and the 3′ end is labeled with TAMRA.

## Data Availability

All data and materials supporting the findings of the current study are available within this article or from the corresponding authors on reasonable request.

## References

[1] Ko G. J., Rhee C. M., Kalantar-Zadeh K., Joshi S. (2020). The effects of high-protein diets on kidney health and longevity. *Journal of the American Society of Nephrology*.

[2] Griffin K. A. (2017). Hypertensive kidney injury and the progression of chronic kidney disease. *Hypertension*.

[3] Howard G., Cushman M., Moy C. S. (2018). Association of clinical and social factors with excess hypertension risk in black compared with white US Adults. *JAMA*.

[4] Jiang X., Liu X., Liu X. (2020). Low-dose aspirin treatment attenuates male rat salt-sensitive hypertension via platelet cyclooxygenase 1 and complement cascade pathway. *Journal of the American Heart Association*.

[5] Tian N., Thrasher K. D., Gundy P. D., Hughson M. D., Manning R. D. (2005). Antioxidant treatment prevents renal damage and dysfunction and reduces arterial pressure in salt-sensitive hypertension. *Hypertension*.

[6] Robinson A. T., Edwards D. G., Farquhar W. B. (2019). The influence of dietary salt beyond blood pressure. *Current Hypertension Reports*.

[7] He F. J., Tan M., Ma Y., MacGregor G. A. (2020). Salt Reduction to Prevent Hypertension and Cardiovascular Disease:. *Journal of the American College of Cardiology*.

[8] Ma Q., Zhao J., Cao W., Liu J., Cui S. (2015). Estradiol decreases taurine level by reducing cysteine sulfinic acid decarboxylase via the estrogen receptor-*α* in female mice liver. *American Journal of Physiology. Gastrointestinal and Liver Physiology*.

[9] Jakaria M., Azam S., Haque M. E. (2019). Taurine and its analogs in neurological disorders: focus on therapeutic potential and molecular mechanisms. *Redox Biology*.

[10] Mozaffari M. S., Abdelsayed R., Patel C., Wimborne H., Liu J., Schaffer S. W. (2010). Differential effects of taurine treatment and taurine deficiency on the outcome of renal ischemia reperfusion injury. *Journal of Biomedical Science*.

[11] Hu J., Xu X., Yang J., Wu G., Sun C., Lv Q. (2009). Antihypertensive effect of taurine in rat. *Advances in Experimental Medicine and Biology*.

[12] Nara Y., Yamori Y., Lovenberg W. (1978). Effect of dietary taurine on blood pressure in spontaneously hypertensive rats. *Biochemical Pharmacology*.

[13] Hagar H. H., El Etter E., Arafa M. (2006). Taurine attenuates hypertension and renal dysfunction induced by cyclosporine A in rats. *Clinical and Experimental Pharmacology & Physiology*.

[14] Inoue A., Takahashi H., Lee L. C. (1988). Retardation of the development of hypertension in DOCA salt rats by taurine supplement. *Cardiovascular Research*.

[15] Sun Q., Wang B., Li Y. (2016). Taurine supplementation lowers blood pressure and improves vascular function in prehypertension: randomized, double-blind, placebo-controlled study. *Hypertension*.

[16] Hosoki R., Matsuki N., Kimura H. (1997). The possible role of hydrogen sulfide as an endogenous smooth muscle relaxant in synergy with nitric oxide. *Biochemical and Biophysical Research Communications*.

[17] Lv B., Chen S., Tang C., Jin H., Du J., Huang Y. (2021). Hydrogen sulfide and vascular regulation - an update. *Journal of Advanced Research*.

[18] Cao X., Bian J. S. (2016). The role of hydrogen sulfide in renal system. *Frontiers in Pharmacology*.

[19] Shibuya N., Koike S., Tanaka M. (2013). A novel pathway for the production of hydrogen sulfide from D-cysteine in mammalian cells. *Nature Communications*.

[20] Kimura H. (2014). The physiological role of hydrogen sulfide and beyond. *Nitric Oxide*.

[21] Zhang Y., Wang Y., Read E. (2020). Golgi stress response, hydrogen sulfide metabolism, and intracellular calcium homeostasis. *Antioxidants & Redox Signaling*.

[22] Wang Y., Yu R., Wu L., Yang G. (2020). Hydrogen sulfide signaling in regulation of cell behaviors. *Nitric Oxide*.

[23] Yan H., Du J., Tang C. (2004). The possible role of hydrogen sulfide on the pathogenesis of spontaneous hypertension in rats. *Biochemical and Biophysical Research Communications*.

[24] Huang P., Chen S., Wang Y. (2015). Down-regulated CBS/H_2_S pathway is involved in high-salt- induced hypertension in Dahl rats. *Nitric Oxide*.

[25] Zhong G., Chen F., Cheng Y., Tang C. S., Du J. B. (2003). The role of hydrogen sulfide generation in the pathogenesis of hypertension in rats induced by inhibition of nitric oxide synthase. *Journal of Hypertension*.

[26] Lu M., Liu Y. H., Goh H. S. (2010). Hydrogen sulfide inhibits plasma renin activity. *Journal of the American Society of Nephrology*.

[27] Li J., Teng X., Jin S. (2019). Hydrogen sulfide improves endothelial dysfunction by inhibiting the vicious cycle of NLRP3 inflammasome and oxidative stress in spontaneously hypertensive rats. *Journal of Hypertension*.

[28] Huang P., Shen Z., Liu J. (2016). Hydrogen sulfide inhibits high-salt diet-induced renal oxidative stress and kidney injury in Dahl rats. *Oxidative Medicine and Cellular Longevity*.

[29] Chen Y., Jin S., Teng X. (2018). Hydrogen sulfide attenuates LPS-induced acute kidney injury by inhibiting inflammation and oxidative stress. *Oxidative Medicine and Cellular Longevity*.

[30] Lin F., Liao C., Sun Y. (2017). Hydrogen sulfide inhibits cigarette smoke-induced endoplasmic reticulum stress and apoptosis in bronchial epithelial cells. *Frontiers in Pharmacology*.

[31] Diaz Encarnacion M. M., Warner G. M., Gray C. E. (2008). Signaling pathways modulated by fish oil in salt-sensitive hypertension. *American Journal of Physiology. Renal Physiology*.

[32] Chen H. Z., Wang F., Gao P. (2016). Age-associated sirtuin 1 reduction in vascular smooth muscle links vascular senescence and inflammation to abdominal aortic aneurysm. *Circulation Research*.

[33] Xu H., Du S., Fang B. (2019). VSMC-specific EP4 deletion exacerbates angiotensin II-induced aortic dissection by increasing vascular inflammation and blood pressure. *Proceedings of the National Academy of Sciences of the United States of America*.

[34] Zong Y., Huang Y., Chen S. (2015). Downregulation of endogenous hydrogen sulfide pathway is involved in mitochondrion-related endothelial cell apoptosis induced by high salt. *Oxidative Medicine and Cellular Longevity*.

[35] Mariem W., Kilani B. R., Benet G. (2014). How does NaCl improve tolerance to cadmium in the halophyte *Sesuvium portulacastrum*?. *Chemosphere*.

[36] Chang L., Xu J. X., Zhao J., Pang Y. Z., Tang C. S., Qi Y. F. (2004). Taurine antagonized oxidative stress injury induced by homocysteine in rat vascular smooth muscle cells. *Acta Pharmacologica Sinica*.

[37] Du J., Huang Y., Yan H. (2014). Hydrogen sulfide suppresses oxidized low-density lipoprotein (Ox- LDL)-stimulated monocyte chemoattractant protein 1 generation from macrophages via the nuclear factor *κ*B (NF-*κ*B) pathway. *The Journal of Biological Chemistry*.

[38] Pei J. F., Li X. K., Li W. Q. (2019). Diurnal oscillations of endogenous H_2_O_2_ sustained by p66^Shc^ regulate circadian clocks. *Nature Cell Biology*.

[39] Jia G., Yu S., Sun W. (2020). Hydrogen sulfide attenuates particulate matter-induced emphysema and airway inflammation through Nrf2-dependent manner. *Frontiers in Pharmacology*.

[40] Stipanuk M. H. (1979). Effect of excess dietary methionine on the catabolism of cysteine in rats. *The Journal of Nutrition*.

[41] Singh P., Rao P., Bhattacharya R. (2013). Dose and time-dependent effects of cyanide on thiosulfate sulfurtransferase, 3-mercaptopyruvate sulfurtransferase, and cystathionine *λ*-lyase activities. *Journal of Biochemical and Molecular Toxicology*.

[42] Luan Z., Wei Y., Huo X. (2021). Pregnane X receptor (PXR) protects against cisplatin-induced acute kidney injury in mice. *Biochimica et Biophysica Acta (BBA) - Molecular Basis of Disease*.

[43] Katakawa M., Fukuda N., Tsunemi A. (2016). Taurine and magnesium supplementation enhances the function of endothelial progenitor cells through antioxidation in healthy men and spontaneously hypertensive rats. *Hypertension Research*.

[44] Lv Q., Yang Q., Cui Y. (2017). Effects of taurine on ACE, ACE2 and HSP70 expression of hypothalamic-pituitary-adrenal axis in stress-induced hypertensive rats. *Advances in Experimental Medicine and Biology*.

[45] Dong G., Zhang L., Tang J., Su J., Tang C. (1992). The effect of taurine on hypertension in rats. *Chinese Pharmacological Bulletin*.

[46] Ideishi M., Miura S., Sakai T., Sasaguri M., Misumi Y., Arakawa K. (1994). Taurine amplifies renal kallikrein and prevents salt-induced hypertension in dahl rats. *Journal of Hypertension*.

[47] Dawson R., Liu S., Jung B., Messina S., Eppler B. (2000). Effects of high salt diets and taurine on the development of hypertension in the stroke-prone spontaneously hypertensive rat. *Amino Acids*.

[48] Chiba Y., Ando K., Fujita T. (2002). The protective effects of taurine against renal damage by salt loading in Dahl salt-sensitive rats. *Journal of Hypertension*.

[49] Lu M., Liu Y. H., Ho C. Y., Tiong C. X., Bian J. S. (2012). Hydrogen sulfide regulates cAMP homeostasis and renin degranulation in As4.1 and rat renin-rich kidney cells. *American Journal of Physiology. Cell Physiology*.

[50] Tan B. L., Norhaizan M. E., Liew W. P. (2018). Nutrients and oxidative stress: friend or foe?. *Oxidative Medicine and Cellular Longevity*.

[51] Marrocco I., Altieri F., Peluso I. (2017). Measurement and clinical significance of biomarkers of oxidative stress in humans. *Oxidative Medicine and Cellular Longevity*.

[52] Li J., White J., Guo L. (2009). Salt inactivates endothelial nitric oxide synthase in endothelial cells. *The Journal of Nutrition*.

[53] Jeffery J., Ayling R. M., McGonigle R. J. (2007). Successful rescue of severe hypernatraemia (196 mmol/L) by treatment with hypotonic fluid. *Annals of Clinical Biochemistry*.

[54] Xu N., Liu S., Lu Z. (2020). Gene expression profiles and flavonoid accumulation during salt stress in Ginkgo biloba seedlings. *Plants*.

[55] Tejera N. A., Ortega E., Gonzalez-Lopez J., Lluch C. (2003). Effect of some abiotic factors on the biological activity of Gluconacetobacter diazotrophicus. *Journal of Applied Microbiology*.

[56] Katz U., Zaccone G., Fasulo S., Mauceri A., Gabbay S. (1997). Lectin binding pattern and band 3 localization in toad skin epithelium and the effect of salt acclimation. *Biology of the Cell*.

[57] Nonaka H., Tsujino T., Watari Y., Emoto N., Yokoyama M. (2001). Taurine prevents the decrease in expression and secretion of extracellular superoxide dismutase induced by homocysteine: amelioration of homocysteine-induced endoplasmic reticulum stress by taurine. *Circulation*.

[58] Bustamante J., Lobo M. V., Alonso F. J. (2001). An osmotic-sensitive taurine pool is localized in rat pancreatic islet cells containing glucagon and somatostatin. *American Journal of Physiology. Endocrinology and Metabolism*.

[59] Hizoh I., Haller C. (2002). Radiocontrast-induced renal tubular cell apoptosis: hypertonic versus oxidative stress. *Investigative Radiology*.

[60] Chang L., Zhao J., Xu J., Jiang W., Tang C. S., Qi Y. F. (2004). Effects of taurine and homocysteine on calcium homeostasis and hydrogen peroxide and superoxide anions in rat myocardial mitochondria. *Clinical and Experimental Pharmacology & Physiology*.

[61] Ueno T., Iguro Y., Yotsumoto G. (2007). Taurine at early reperfusion significantly reduces myocardial damage and preserves cardiac function in the isolated rat heart. *Resuscitation*.

[62] Zeng K., Xu H., Mi M. (2010). Effects of taurine on glial cells apoptosis and taurine transporter expression in retina under diabetic conditions. *Neurochemical Research*.

[63] Zeng K., Xu H., Chen K. (2010). Effects of taurine on glutamate uptake and degradation in Muller cells under diabetic conditions via antioxidant mechanism. *Molecular and Cellular Neurosciences*.

[64] Lin S., Wu G., Zhao D. (2017). Taurine increases insulin expression in STZ-treated rat islet cells in vitro. *Advances in Experimental Medicine and Biology*.

[65] Trachtman H., Del Pizzo R., Rao P., Rujikarn N., Sturman J. A. (1989). Taurine lowers blood pressure in the spontaneously hypertensive rat by a catecholamine independent mechanism. *American Journal of Hypertension*.

[66] Adedara I. A., Alake S. E., Olajide L. O., Adeyemo M. O., Ajibade T. O., Farombi E. O. (2019). Taurine ameliorates thyroid hypofunction and renal injury in L-NAME-induced hypertensive rats. *Drug Research*.

[67] Nandhini A. T., Anuradha C. V. (2004). Hoe 140 abolishes the blood pressure lowering effect of taurine in high fructose-fed rats. *Amino Acids*.

[68] Feng Y., Sun F., Gao Y. (2017). Taurine decreased uric acid levels in hyperuricemic rats and alleviated kidney injury. *Biochemical and Biophysical Research Communications*.

[69] Yao H. T., Lin P., Chang Y. W. (2009). Effect of taurine supplementation on cytochrome P450 2E1 and oxidative stress in the liver and kidneys of rats with streptozotocin-induced diabetes. *Food and Chemical Toxicology*.

[70] Murakami S., Sakurai T., Toda Y., Morito A., Sakono M., Fukuda N. (2010). Prevention of neointima formation by taurine ingestion after carotid balloon injury. *Vascular Pharmacology*.

